# Using science to sell apps: Evaluation of mental health app store quality claims

**DOI:** 10.1038/s41746-019-0093-1

**Published:** 2019-03-22

**Authors:** Mark Erik Larsen, Kit Huckvale, Jennifer Nicholas, John Torous, Louise Birrell, Emily Li, Bill Reda

**Affiliations:** 1 Black Dog Institute, University of New South Wales, Sydney, NSW Australia; 2 Center for Behavioral Intervention Technologies, Northwestern University, Chicago, IL USA; 3 Division of Digital Psychiatry, Beth Israel Deaconess Medical Centre, Harvard Medical School, Boston, MA USA; 4 Centre for Research Excellence in Mental Health and Substance Use, National Drug and Alcohol Research Centre, University of New South Wales, Sydney, NSW Australia

**Keywords:** Translational research, Psychiatric disorders, Public health

## Abstract

Despite the emergence of curated app libraries for mental health apps, personal searches by consumers remain a common method for discovering apps. App store descriptions therefore represent a key channel to inform consumer choice. This study examined the claims invoked through these app store descriptions, the extent to which scientific language is used to support such claims, and the corresponding evidence in the literature. Google Play and iTunes were searched for apps related to depression, self-harm, substance use, anxiety, and schizophrenia. The descriptions of the top-ranking, consumer-focused apps were coded to identify claims of acceptability and effectiveness, and forms of supporting statement. For apps which invoked ostensibly scientific principles, a literature search was conducted to assess their credibility. Seventy-three apps were coded, and the majority (64%) claimed effectiveness at diagnosing a mental health condition, or improving symptoms, mood or self-management. Scientific language was most frequently used to support these effectiveness claims (44%), although this included techniques not validated by literature searches (8/24 = 33%). Two apps described low-quality, primary evidence to support the use of the app. Only one app included a citation to published literature. A minority of apps (14%) described design or development involving lived experience, and none referenced certification or accreditation processes such as app libraries. Scientific language was the most frequently invoked form of support for use of mental health apps; however, high-quality evidence is not commonly described. Improved knowledge translation strategies may improve the adoption of other strategies, such as certification or lived experience co-design.

## Introduction

Recent reviews have found mobile health (mHealth) apps to be effective in reducing symptoms of depression^1^ and anxiety;^2^ however, authors acknowledge the disparity between apps with research evidence and the apps currently available to – and used by – consumers. Reviews of the quality of the content within publicly available health apps^3,4^ and specifically mental health apps^5–7^ support this disparity, reporting that the majority of consumer-available apps are not evidence-based and can contain harmful content.

Although there is an increasing interest in accreditation processes,^8^ app libraries^9,10^ and frameworks to support clinicians in recommending mental health apps,^11^ personal searches on commercial app stores operated by the major smartphone platform providers remain a common method for discovering mental health apps.^12^ In this setting, marketing materials provided by developers are a principal source of information to inform consumer or clinician choice. The format of this material is standardised for commercial app stores, consisting of a written app description and, optionally, screenshots or videos of app functions.

Within this restricted context, the extent to which scientific evidence is presented as a potential marker of quality for health apps is unclear. A preliminary investigation by the authors previously reported that, for apps clinically relevant for depression, 38% of app store descriptions included wording related to claims of effectiveness, whereas only 2.6% provided evidence to substantiate such claims.^13^

This study aims to extend this preliminary analysis to further understand how scientific evidence is currently used to market and sell mental health apps by (i) examining the types of claims made by mental health apps and, specifically, estimating the proportion of apps that invoke claims of effectiveness; (ii) describing the types of supporting statements used to justify claims and, specifically, estimating the proportion of apps which invoke scientific principles; and (iii) assessing the credibility of scientific principles that are used as supporting statements. Insight into methods used to present apps on commercial stores has the potential to inform government and professional efforts to establish curated libraries for health apps, as well as develop our understanding of translational gaps between mHealth research and developer practices.

## Results

### Search and screening

A total of 1435 apps were identified through searches of the app stores (see Table 1). Three hundred and fifty apps were screened for eligibility – representing the top 40 ranked apps in each search, except where fewer iOS apps were returned for schizophrenia, self-harm and substance use. Inter-rater reliability for the binary choice to include or exclude each app was measured using Cohen’s kappa at 0.78, suggesting moderate agreement. Following screening for eligibility and removal of duplicates across search terms and platforms, 76 platform-independent apps were retained for coding. During the coding process, an additional three apps were identified as being targeted at clinicians or health professionals; excluding these apps resulted in 73 apps being retained for full coding.

**Table 1:** Number of apps identified and screened for eligibility

Search term	Identified in searches (*n* = 1435)		Screened (*n* = 350)	
	Android	iOS	Android	iOS
Anxiety	249	200	40	40
Depression	250	200	40	40
Schizophrenia	250	32	40	32
Self-harm	85	29	40	29
Substance use	131	9	40	9
Total	965	470	200	150

### App functionality

The majority of apps (59/73, 81%) described a single mental health-related functionality; fewer apps described two (8/73, 11%) or three (3/73, 4.1%) discrete functions. Three apps did not clearly describe any specific functionality (3/73, 4.1%). The types of functionality described by the apps are summarised in Table 2.

**Table 2:** Functionality of apps included in the review

Functionality	*n* (%) of apps
§2.i. Self-assessment	9 (12)
§2.ii. Symptom or mood monitoring	18 (25)
§2.iii. Information or psychoeducation	26 (36)
§2.iv. Therapy or treatment	23 (32)
§2.v. Peer or community support	8 (11)

The total exceeds 100% due to apps describing multiple functionalities

### Claims and disclaimers

Just over four-fifths of apps (§3, 59/73, 81%) made a positive claim in their online app store description, including claims related to effectiveness (§3.a, 47/73, 64%) or acceptability (§3b, 33/73, 45%) – see Table 3. Twenty-one of these apps claimed both effectiveness and acceptability. The most common form of effectiveness claim was related to improvements in knowledge or skills to support self-management (§3.a.iii, 26/73, 36%), closely followed by improvements in symptoms or mood (§3.a.ii, 22/73, 30%), with fewer apps claiming the ability to diagnose or detect a mental health condition (§3.a.i, 7/73, 10%). A subset of eight apps (8/73, 11%) claimed both improvements in self-management and symptoms. Just under one-third of apps (§5, 22/73, 30%) included some form of disclaimer – either a medical disclaimer (§5.a, 20/73, 27%) or less commonly a legal disclaimer (§5.b, 8/73, 11%).

**Table 3:** Number of apps with positive claims, supporting statements, and disclaimers in their app store descriptions

Coding element	*n* (%) of apps
§3. Positive claims	59 (81)
§3.a. Claims of effectiveness	47 (64)
§3.a.i. Detection or diagnosis	7 (10)
§3.a.ii. Improvement in symptoms or mood	22 (30)
§3.a.iii. Improvement in self-management	26 (36)
§3.b. Claims of acceptability	33 (45)
§4. Supporting statements	47 (64)
§4.a. Scientific language	32 (44)
§4.a.i. Specific technique described	24 (33)
§4.a.ii. Evidence from study using app	2 (2.7)
§4.a.iii. Citation to scientific literature	1 (1.4)
§4.b. Technical expertise	23 (32)
§4.b.i. Certification or accreditation	0
§4.b.ii. Prizes or awards	2 (2.7)
§4.b.iii. Credible developers	18 (25)
§4.b.iv. Credible endorsements	3 (4.1)
§4.c. Lived experience design	10 (14)
§4.c.i. Lived experience involvement	6 (8.2)
§4.c.ii. Lived experience developer	5 (6.8)
§4.d. “Wisdom of the crowd”	14 (19)
§4.d.i. Download, usage or popularity statistics	11 (15)
§4.d.ii. User testimonials	8 (11)
§4.d.iii. Press endorsements	6 (8.2)
§5. Negative claims	22 (30)
§5.a. Medical disclaimer	20 (27)
§5.b. Legal disclaimer	8 (11)

All percentages are reported based on *n* = 73

### Supporting statements

Forty-seven apps (§4, 47/73, 64%) also provided some form of statement supporting use of the app (although this is the same number as provided claims of effectiveness, this represents a different, but overlapping, set of apps). The most common form of support was the use of scientific language (§4.a, 32/73, 44%), although eight of these apps used general terms (e.g. “evidence-based treatment”); specific scientific methods or techniques were identified for 24 apps (§4.a.i, 24/73, 33%) – full details of the annotated techniques are described later. Notably only two apps (§4.a.ii, 2/73, 2.7%) described direct evidence associated with the app (a description of a pilot study reducing symptoms of anxiety and depression, and data indicating users regularly report feeling better after using the app), and only one app (§4.a.iii, 1/73, 1.4%) provided citation details to scientific literature (a validation paper associated with a self-report questionnaire). A post-hoc analysis identified that five apps (5/73, 6.8%) mentioned research or clinical trials underway.

The second most common type of support was the description of technical expertise (§4.b, 23/73, 32%). This was predominantly through descriptions of the credibility of the app developer (§4.b.iii, 18/73, 25%), and less commonly through inclusion of expert endorsements (§4.b.iv, 3/73, 4.1%) or awards and prizes (§4.b.ii, 2/73, 2.7%). No apps referred to formal accreditation or certification schemes (§4.b.i).

Ten apps (§4.c, 10/73, 14%) referred to lived experience perspectives, either in their design or development process (§4.c.i, 6/73, 8.2%) or in the development team itself (§4.c.ii, 5/73, 6.8%). App descriptions invoked the “wisdom of the crowd” in just under one-fifth of cases (§4.d, 14/73, 19%), referring to download, usage, or popularity metrics (§4.d.i, 11/73, 15%), user testimonials and reviews ($4.d.ii, 8/73, 11%), or press endorsements (§4.d.iii, 6/73, 8.2%).

### Effectiveness claims and their supporting statements

**Fig. 1:**
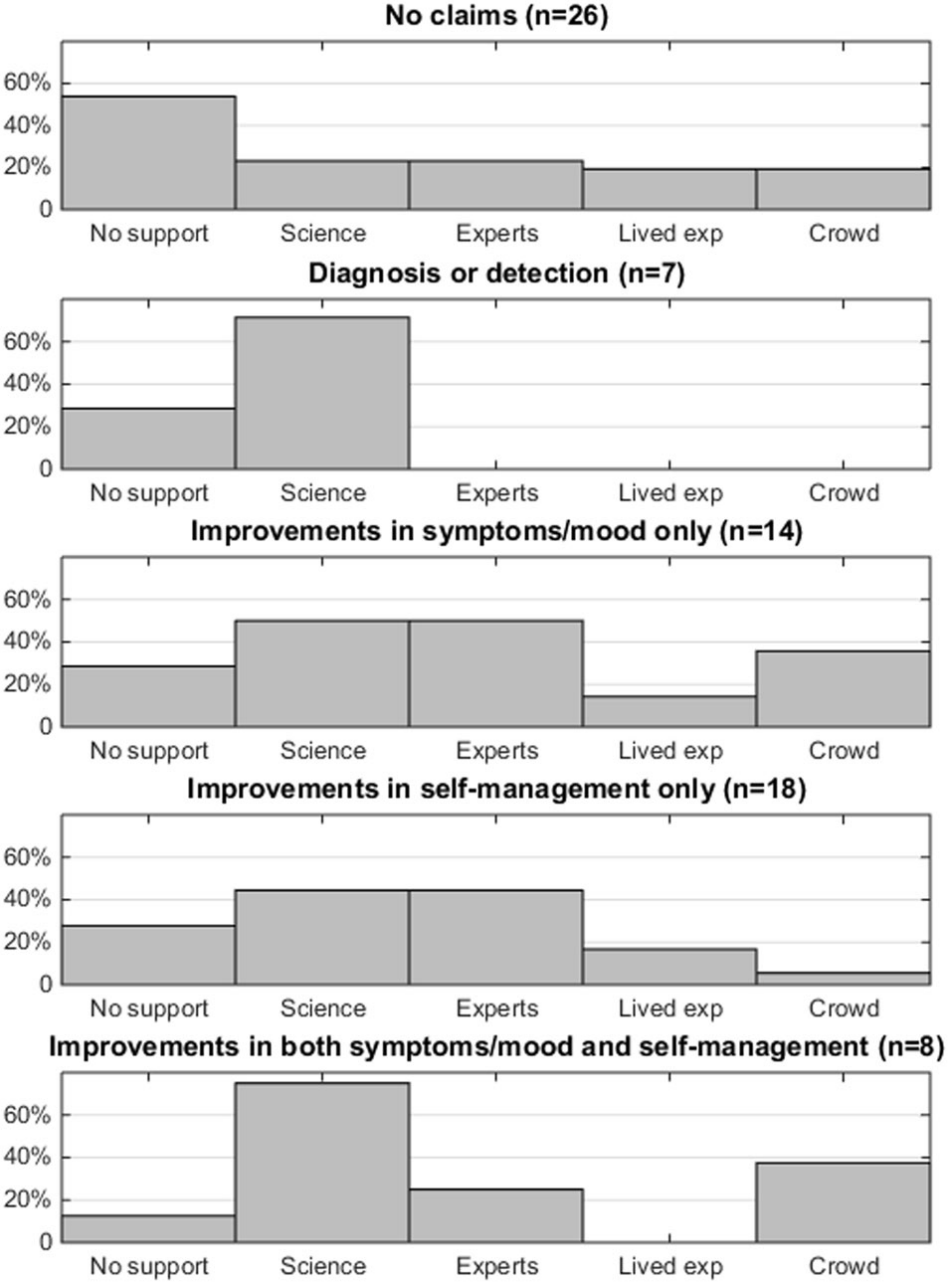
Histograms showing the frequency of specific categories of supporting statements based on the type of effectiveness claim made by an app. Each app can contain multiple types of supporting statements

Apps were grouped together based on the type of effectiveness claims made, and the associated supporting statements were examined – see Fig. 1. The largest single category was apps that did not make a claim of effectiveness (*n* = 26), of which just over half (14/26, 54%) also did not include supporting statements. However, where supporting statements were included, these were evenly distributed across the categories. The small number of apps which made claims related to diagnosis or detection of a mental health condition exclusively invoked supporting statements related to scientific language (*n* = 5/7, 71%).

Approximately half of the apps included a single type of claim related to improvements in symptoms or self-monitoring. In this set of apps, scientific language and descriptions of technical expertise were invoked equally. For the set of apps that claimed improvements in both symptoms and self-management, supporting statements were predominantly related to scientific statements (*n* = 6/8, 75%) and to the exclusion of statements about lived experience involvement.

### App functionality and supporting statements

**Fig. 2:**
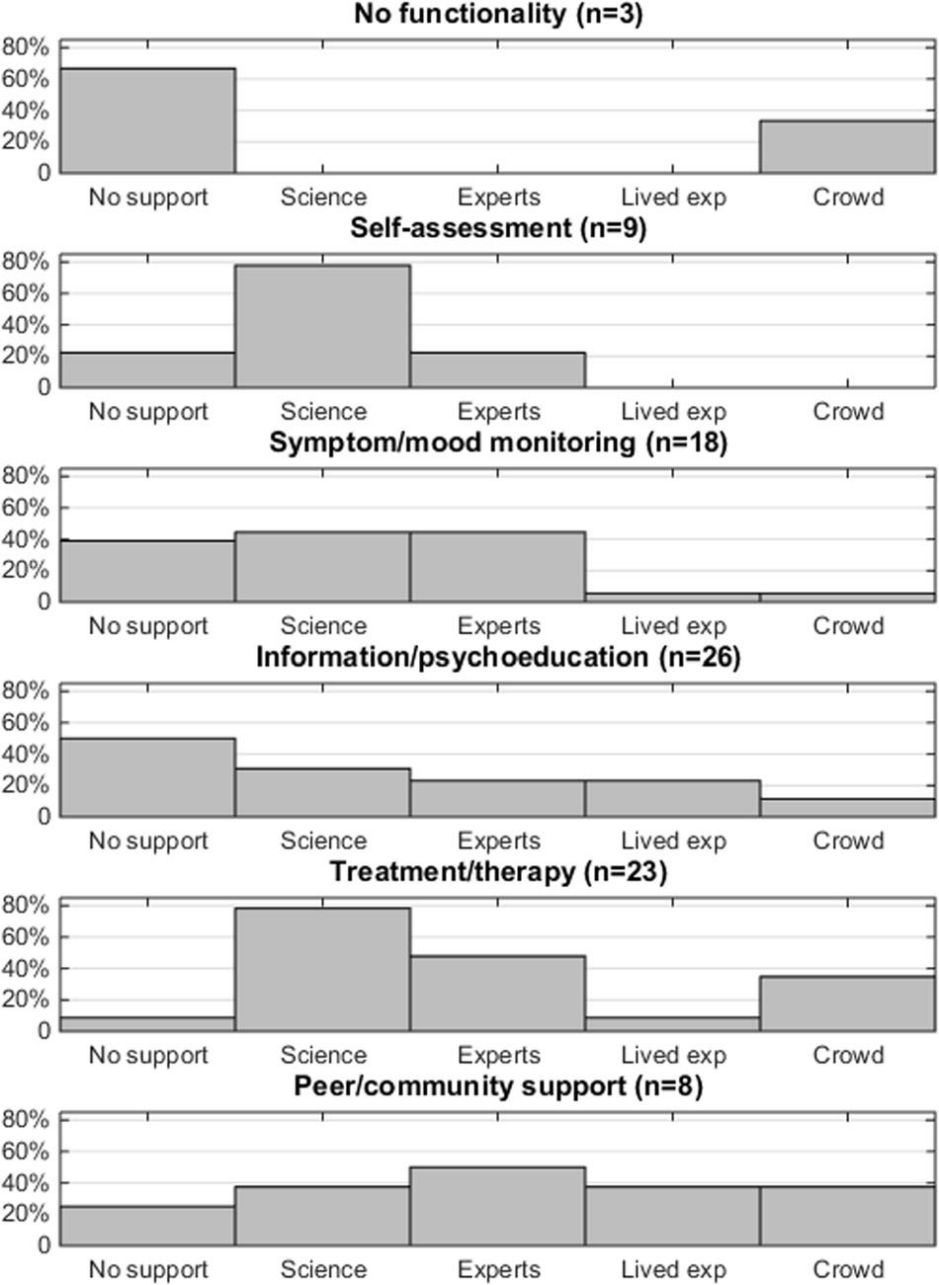
Histograms showing the frequency of specific categories of supporting statements based on the app functionality. Each app can contain multiple functionalities, and multiple types of supporting statements

Apps were also grouped together based on the functionality of the app, and the types of supporting statements invoked were examined – see Fig. 2. The most common app functionality was to provide information or psychoeducational content, and half (*n* = 13/26, 50%) of these apps provided no supporting statements. Scientific language was frequently used in apps for treatment or therapy (*n* = 18/23, 78%) or self-assessment (*n* = 7/9, 78%). Apps involving peer-support or community support included the highest proportion of support involving technical expertise (*n* = 4/8, 50%), lived experience perspectives (*n* = 3/8, 38%) and the wisdom of the crowd (*n* = 3/8, 38%).

### Evidence search

From the descriptions of the 24 apps which mentioned a specific scientific technique, 11 unique conditions (§1) and 38 unique methods (§3.a.i) were identified, resulting in 49 unique literature searches being conducted – the results of which are presented in Supplementary Information 1. The most frequent combination found was the mention of cognitive behavioural therapy in relation to depression and anxiety (*n* = 7 and *n* = 6, respectively), for which positive evidence was found.^14^ The second most common combination was the use of “binaural beats” in relation to depression and anxiety (*n* = 4 and *n* = 3, respectively), for which no evidence could be found in the scientific literature. Other combinations described more than once were generally associated with positive evidence, including dialectical behaviour therapy for self-harm (*n* = 3),^15^ the use of the Patient Health Questionnaire (PHQ-9) for the assessment of depression (*n* = 3),^16^ the Generalised Anxiety Disorder (GAD-7) questionnaire for the assessment of anxiety (*n* = 2),^17^ and a harm reduction approach in substance use (*n* = 2).^18^ Active listening was also mentioned in reference to a range of conditions, and although no specific evidence could be identified in the literature searches, it is acknowledged that this is considered a key clinical skill.^19^ From the remaining combinations of techniques and conditions which were described once, the majority were also associated with positive evidence (*n* = 20), and the remainder with unclear evidence (*n* = 9) or no found evidence (*n* = 8).

Overall, from the 49 combinations of conditions and methods, 26 (53%) were associated with positive evidence, 13 (27%) were associated with unclear evidence, and evidence could not be found for 10 (20%). Aggregating at an app-level, a third of the apps described at least one technique for which evidence could not be found (8/24, 33%).

## Discussion

Seventy-three mental health apps, representing the most highly ranked apps from the two major app stores, were examined in this study. Sixty-four percent of these apps made positive claims about their effectiveness, and 45% claimed acceptability. Statements supporting the use of the apps were presented through scientific descriptions (44%), technical expertise (32%), appeals to the “wisdom of the crowd” (19%), or lived experience involvement (14%). Of the scientific methods described, just over a half (53%) were associated with evidence in academic literature; of the apps describing specific scientific techniques, a third referred to techniques for which no evidence could be found (33%).

From a research perspective, it is perhaps reassuring that scientific language was the leading form of support employed by developers; however, this was present in fewer than half of the apps. Importantly, only two apps (2.7%) provided direct evidence associated with app use – results from a pilot study, and user-reported changes in mood after app use. One app description (1.3%) cited a validation paper for a self-report questionnaire. While these cases represent the best evidence provided by apps in this study, they still fall short of high-quality evidence obtained, for example, from randomised controlled trials.

Although there may be a lack of published evidence directly supporting the use of the mental health apps examined in this review, when apps described scientific techniques more broadly, in just over half the cases these techniques were associated with good evidence from the literature. This raises the hope that apps are evidence-informed, if not necessarily evidence-based. Caution, however, is still required as apps claiming to deliver, for example, cognitive behavioural therapy for depression may have minimal concordance with the actual principles of CBT.^20^ Furthermore, a third of apps whose descriptions included scientific techniques referred to principles that had no evidence available in the scientific literature. Together with those apps which cited principles with conflicting evidence, and those which used general scientific language without reference to specific methods, this suggests that developers are using scientific language to appeal to consumers, regardless of the accuracy of the claims. Sector engagement with app developers and consumers may help improve the reporting and understanding of the science associated with mental health apps.

These results are also important in the context of new efforts to regulate health apps. The United States Food and Drug Administration (FDA) is exploring a Software Precertification (Pre-Cert) Pilot Program that will shift regulation towards the app manufacturers themselves and rely on “monitoring real-world performance” of apps in the wild.^8^ Given the variable quality of evidence identified in this study, this suggests there may be an opportunity for researchers to work with developers to identify how high-quality evidence and real-world performance data could best be captured.

Of the categories of supporting statements identified in this study, the least frequently described was the involvement of those with lived experience (14%). It is acknowledged that consumer involvement and co-design of interventions can be a key factor for their success,^21,22^ and conversely a lack of involvement is often associated with poor uptake and engagement of digital interventions.^23^ These factors highlight the potential for increased lived experience involvement in the development of mental health apps.

It was also noted that despite increasing interest in app accreditation frameworks and curated libraries, no apps described these in their app store descriptions. For the apps in this study, which already have good visibility through high search result rankings, this may reflect a lack of perceived need for such processes. It may alternatively reflect a lack of awareness of these schemes outside academic or clinical communities, or that accreditation could be used as a marker of credibility in a commercial marketplace. Regardless of the underlying reason, further knowledge translation activities appear to be warranted to increase the profile of such accreditation schemes. One such scheme attempting to identify quality apps for clinical and individual use is the American Psychiatric Association (APA) app evaluation scheme.^11^ Although the APA app evaluation framework does not offer direct recommendations or marks of approval, it focuses on informed decision making and helps clinicians and individuals consider the risks and benefits of app use on a case-by-case basis. Such an approach supports selection of an app based upon the individual needs of a user, with clinician consideration of the scientific claims and evidence associated with the app. Future reviews may be warranted to examine whether references to accreditation schemes increase with the adoption of schemes such as the APA framework and the implementation of FDA pre-cert.

In the future, app stores could include standardised data fields allowing developers to provide additional details to support their apps. There has been progress towards mandating that apps include a privacy policy, and this could be extended for health apps by allowing developers to include a PubMed identifier, offering users the opportunity to click through to published articles related to the app,^13^ as well as other indicators such as compliance with quality frameworks and lived experience involvement.

It is acknowledged that this study provides only a snapshot of a subset of mental health apps, and that the app stores represent a rapidly evolving ecosystem for distribution of health apps.^13^ Nevertheless, these results provide a broad indication of the nature and credibility of claims associated with mental health apps. The study did not examine either the content of ancillary marketing material presented alongside app descriptions, such as screenshots or user comments. These elements were excluded partly for reasons of standardisation (as all apps include a structured textual description but may not include other elements) and partly because it was considered unlikely that either imagery or user comments would reference scientific principles, which was a key purpose of this study. Previous research indicates that while users provide a range of positive or negative ratings, there is only minimal mention of scientific quality or evidence.^24^

At the outset of this study, we initially aimed to differentiate between claims related to improvements in mood and improvements in symptoms, as a means of differentiating, for example, feelings of depression vs symptoms of clinical depression. However, it became apparent that such distinctions were not clearly articulated within the app store descriptions, so these coding categories were combined.

This study included appeals to the “wisdom of the crowd” and lived experience involvement as markers of credibility which can be used to support claims made in app store descriptions. It should be noted, however, that user ratings do not necessarily correlate well with clinical utility or quality.^6,25^

Scientific methods are reported in this study using a three-point evidence scale. More rigorous evidence evaluation schemes exist, for example, through formal systematic review, meta-analysis and the OCEBM Levels of Evidence^26^ – however, such a rigorous approach was not possible here due to the number of literature searches required (*n* = 49). Nevertheless, the three-point scale incorporated existing systematic reviews, where available, to differentiate techniques for a particular mental health condition for which there is clear evidence in the literature, mixed or unclear evidence, or no evidence found. Further inspection of in-app content, by multiple stakeholders including those with lived experience and clinical expertise, would be required to obtain a complete understanding of the quality of an app. This would also include an assessment of whether the scientific principles cited are actually used within the app, and to what degree of fidelity.

This review has examined a set of markers of quality which can be derived from app store descriptions, with a particular focus on the description of scientific techniques and evidence. However, these are not the only important markers of quality. Additional factors such as usability, data privacy and security, and integration with clinical workflows and systems are also of importance, and may not be discernible from just the app store description. These domains are included in guidelines for the relaunched NHS Apps Library^27^ and the APA framework,^11^ amongst others, and serve as a best-practice guide in terms of app development standards and important information to be provided to allow individuals, clinicians or app library providers to make informed decisions about app adoption.

This study examined 73 of the top ranked mental health apps publicly available to map the nature of claims and the type of supporting statements employed in app descriptions presented in app stores. Scientific language was the most frequently employed strategy for supporting effectiveness claims. However, direct evidence from app-specific studies was lacking, and many apps described techniques for which there was not clear evidence in the literature. Lived experience involvement and engagement with formal accreditation processes were limited, suggesting further knowledge translation activities may be required to raise the awareness of these critical aspects of mental health app development.

## Methods

### Search strategy

Apps were selected for mental health conditions based upon the greatest global burden of disease. Based upon estimates of disability-adjusted life years (DALYs) provided by Vigo et al., the five greatest burdens of disease were considered to be depression, self-harm, substance use disorders (combining drug use disorders and alcohol use disorders), anxiety disorders and schizophrenia.^28^ Chronic pain syndrome was not included due to the uncertainty associated with the allocation of DALYs between mental health and musculoskeletal conditions. Searches for these five conditions (“depression”, “self harm”, “substance use”, “anxiety” and “schizophrenia”) were performed on 21 November 2017. Searches for Android apps were performed on the US Google Play store website, and for iOS apps through the iTunes search application programming interface (API) set to the US store. For each search term on each platform, the app title and description were extracted (manually for Android, and programmatically for iOS) for the top 40 search results.

### Screening

After extracting the search result data, the title and descriptions of each app were reviewed to assess eligibility, using the criteria in Table 4. Apps were not screened at this stage based upon their content nor any claims of effectiveness. Apps were reviewed independently by two coders, with disagreements resolved by discussion to achieve consensus. The consensus set of the top 10 ranked apps for each search term on each platform (according to the order returned by each app store) were retained. Apps which were identified by multiple search terms or across both platforms were de-duplicated.

**Table 4:** Eligibility criteria for identifying the mental health related apps

Inclusion criteria	Exclusion criteria
Apps explicitly related to mental health (including, but not limited to, information, screening, treatment) or emotional states associated with mental health conditions (such as feeling depressed, or feeling anxious).	Apps targeting health professionals, including medical students and conferences.
Apps broadly related to mental health or apps for specific mental health conditions, not limited to the five conditions in the search terms.	Apps not in English.
Apps targeting the public, individuals with a possible mental health condition, or their friends and family.	

### Identification of claims and supporting statements

Two coders annotated each app description using the coding scheme described below. Disagreements were resolved by a third coder. The broad coding categories were defined in advance, with iterative refinements to sub-categories following pilot testing with a subset of the apps. Screenshots and other materials presented on the app stores (e.g. user comments) were not reviewed and are not included in this analysis.

*§1. Target condition(s)*: any mood state or mental health condition identified in the description text was annotated as a target condition for the app. If no conditions were explicitly mentioned, the search term (or terms) which identified the app was used.

*§2. App functionality*: the described function of the app was coded as providing (i) self-assessment; (ii) symptom or mood monitoring; (iii) information or psychoeducation; (iv) therapy or treatment; or (v) peer-support or community support. Zero, one, or more functionalities could be coded.

*§3. Positive claims*: two broad, non-mutually exclusive, categories of positive claims were identified from the app store descriptions:

- *Claims of effectiveness*. Specifically, text was coded as a claim if it linked the use of the app to any of the following outcomes: (i) the detection or diagnosis of a condition; (ii) improvement in symptoms or mood; or (iii) improvement in the individual’s ability to self-manage their condition (for example, through the acquisition of knowledge or skills).
- *Claims of acceptability*, such as statements focusing on the usability or acceptability of the app, rather than the app’s impact on health and wellbeing.

*§4. Supporting statements*: to identify the types of statements used to support the use of the app or the claims made, the following categories were identified:

- *Support invoking scientific language*, specifically: (i) mentions or use of a specific scientific technique, method, or principle; (ii) evidence from a study evaluating use of the app; or (iii) citations to scientific literature. Specific scientific techniques were coded and the perceived credibility or evidence associated with these methods was later evaluated (see §6 – Evidence base, below).
- *Support based on technical expertise*, specifically: (i) any formal quality assessment framework, or certification or accreditation programmes related to the developer or app; (ii) prizes or awards for the developer or the app; (iii) the credibility of the app developer or other professionals associated with the app; or (iv) endorsements from credible or trustworthy professionals or organisations.
- Support based on design informed by lived experience, specifically: (i) involvement of individuals with lived experience in the design or development of the app (including focus group feedback); or (ii) developers with lived experience.
- *Support based on “the wisdom of the crowd”*, specifically: (i) download, usage, or popularity statistics; (ii) testimonials from users; or (iii) endorsements from the press or media.

*§5*. *Negative claims*: within app store descriptions two types of disclaimers were identified (a) medical disclaimers, such as not being a replacement for medical care, and (b) legal disclaimers.

*§6. Evidence base*: coded as either: (a) positive evidence from at least one systematic review or randomised controlled trial, with consensus amongst the reviewers; (b) unclear evidence, where some evidence was found but there was also contradictory evidence identified, concerns about the quality of the evidence, or there was not a clear consensus; or (c) no evidence found, where evidence from a systematic review or randomised controlled trial could not be found. For details of the method used to identify evidence, see the Evidence search section, below.

### Evidence search

After initial coding, each combination of identified target condition (§1) and scientific technique (§4.a.i) were enumerated. A literature search was conducted to try to establish the state of the evidence, if any, supporting the application of each technique to each identified condition. Given the large number of combinations of techniques and conditions, it was not feasible to conduct a full systematic review or meta-analysis for each. Therefore, for pragmatic reasons, searches were conducted using the MEDLINE, Embase, and PsycINFO databases for articles including the combination of technique and condition, limited to either systematic reviews or randomised controlled trials. Two researchers independently performed each search, and reviewed the titles and abstracts, and full-texts where necessary, to determine whether there was evidence found from at least one systematic review or randomised control trial to support the application of a method for a specific condition. Coding disagreements were resolved by a third reviewer. As this may result in a permissive stance, where a positive single randomised controlled trial could be coded as evidence supporting a technique, the resolved coding decisions were then reviewed by an additional two expert coders to identify any relevant literature supporting, or contradicting, the coding. Evidence was summarised using the three-point coding scale described previously in the coding schema.

### Data analysis

Descriptive statistics were used to summarise the results of coding. Sub-group analyses were performed to examine the types of supporting statements invoked for different categories of effectiveness claims, and for different app functionalities.

## Supplementary information

Supplementary Information 1.

Coded app data

## Data Availability

The data supporting the findings of this study are available within the paper and its Supplementary Information files.

## Supplementary information

**Supplementary information** accompanies the paper on the *npj Digital Medicine* website (10.1038/s41746-019-0093-1).
