# Supplementary material for: Using science to sell apps: Evaluation of mental health app store quality claims
Source: NPJ Digit Med. 2019 Mar 22;2:18. doi: 10.1038/s41746-019-0093-1 (PMC6550255; doi:10.1038/s41746-019-0093-1)
Supplement: Supplementary file 1 — Supplementary Information 1. [file 41746_2019_93_MOESM1_ESM.pdf]

Supplementary Information 1

Summary of literature review searches to support methods described in app store descriptions.

**Method**	**Condition/s**	**Number of apps**	**Evidence**
Acceptance and commitment therapy	Anxiety, depression	1	Positive evidence[29]
Active listening	Anxiety, depression, eating disorders, panic disorder	2	Unclear evidence
Behavioural activation	Anxiety	1	Unclear evidence
	Depression	1	Positive evidence[30]
Binaural beats	Anxiety	3	No evidence found
	Depression	4	No evidence found
Brainwave frequencies	Anxiety	1	No evidence found
Breathing exercises	Anxiety, depression	1	Unclear evidence
CAGE questionnaire	Alcohol use	1	Positive evidence[31]
Cognitive behavioural therapy	Agoraphobia	1	Positive evidence[14]
	Anxiety	6	Positive evidence[14]
	Bipolar disorder	1	Positive evidence[32]
	Depression	7	Positive evidence[14]
	Eating disorders	1	Positive evidence[33]
	Panic disorder	1	Positive evidence[14]
	Schizophrenia	1	Positive evidence[34]
Cognitive therapy	Anxiety	1	Positive evidence[35]
	Specific phobia	1	Positive evidence[36]
	Depression	1	Positive evidence[37]
Colour emotion synesthesia	Panic disorder	1	No evidence found

**Method**	**Condition/s**	**Number of apps**	**Evidence**
Dialectical behaviour therapy	Anxiety	1	No evidence found
	Depression	1	Unclear evidence
	Self-harm	3	Positive evidence[15]
Dream therapy	Schizophrenia	1	No evidence found
GAD-7	Anxiety	2	Positive evidence[17]
Harm reduction approach	Substance use	2	Positive evidence[18]
Microactions	Anxiety, depression	1	No evidence found
Mindfulness	Anxiety, depression	1	Positive evidence[38]
Motivational interviewing	Anxiety, depression	1	Positive evidence[39]
Peer-to-peer support	Agoraphobia	1	No evidence found
	Anxiety	1	Positive evidence[40]
	Bipolar disorder, depression, schizophrenia	1	Unclear evidence
PHQ-9	Depression	3	Positive evidence[16]
Positive psychology	Anxiety	1	Positive evidence[41]
	Depression	1	Positive evidence[42]
Psychoeducation	Substance use	1	Positive evidence[43]
Sounds waves	Schizophrenia	1	No evidence found
Yoga	Anxiety, depression	1	Unclear evidence

Additional References

29. Hacker, T., Stone, P. & MacBeth, A. Acceptance and commitment therapy - do we know enough? Cumulative and sequential meta-analyses of randomized controlled trials. *J. Affect Disord.* **190**, 551–565 (2016).

30. Ekers, D. et al. Behavioural activation for depression; an update of meta-analysis of effectiveness and sub group analysis. *PLoS ONE* **9**, e100100 (2014).

31. Dhalla, S. & Kopec, J. A. The CAGE questionnaire for alcohol misuse: a review of reliability and validity studies. *Clin. Invest. Med.* **30**, 33–41 (2007).

32. Chiang, K. J. et al. Efficacy of cognitive-behavioral therapy in patients with bipolar disorder: a meta-analysis of randomized controlled trials. *PLoS ONE* **12**, e0176849 (2017).

33. Linardon, J., Wade, T. D., de la Piedad Garcia, X. & Brennan, L. The efficacy of cognitive-behavioral therapy for eating disorders: a systematic review and metaanalysis. *J. Consult. Clin. Psychol.* **85**, 1080–1094 (2017).

34. Jauhar, S. et al. Cognitive-behavioural therapy for the symptoms of schizophrenia: systematic review and meta-analysis with examination of potential bias. *Br. J. Psychiatry.* **204**, 20–29 (2014).

35. Chiesa, A. & Serretti, A. Mindfulness based cognitive therapy for psychiatric disorders: a systematic review and meta-analysis. *Psychiatry Res.* **187**, 441–453 (2011).

36. Choy, Y., Fyer, A. J. & Lipsitz, J. D. Treatment of specific phobia in adults. *Clin. Psychol. Rev.* **27**, 266–286 (2007).

37. Dobson, K. S. A meta-analysis of the efficacy of cognitive therapy for depression. *J. Consult. Clin. Psychol.* **57**, 414–419 (1989).

38. Hofmann, S. G., Sawyer, A. T., Witt, A. A. & Oh, D. The effect of mindfulness-based therapy on anxiety and depression: A meta-analytic review. *J. Consult. Clin. Psychol.* **78**, 169–183 (2010).

39. Baker, A. L., Thornton, L. K., Hiles, S., Hides, L. & Lubman, D. I. Psychological interventions for alcohol misuse among people with co-occurring depression or anxiety disorders: a systematic review. *J. Affect Disord.* **139**, 217–229 (2012).

40. Ellis, L., Campbell, A., Sethi, S. & O'Dea, B. Comparative randomized trial of an online cognitive-behavioral therapy program and an online support group for depression and anxiety. *J. Cyber. Rehabil.* **4**, 461–467 (2011).

41. Fava, G. A. et al. Well-being therapy of generalized anxiety disorder. *Psychother. Psychosom.* **74**, 26–30 (2005).

42. Bolier, L. et al. Positive psychology interventions: a meta-analysis of randomized controlled studies. *BMC Public Health* **13**, 119 (2013).

43. Kaminer, Y., Burleson, J. A. & Goldberger, R. Cognitive-behavioral coping skills and psychoeducation therapies for adolescent substance abuse. *J. Nerv. Ment. Dis.* **190**, 737–745 (2002).
